# The role of prognostic stratification on prescription of anticoagulants in older patients with atrial fibrillation: a multicenter, observational, prospective European study (EUROSAF)

**DOI:** 10.1080/07853890.2022.2117407

**Published:** 2022-09-05

**Authors:** Alberto Pilotto, Nicola Veronese, Maria Cristina Polidori, Timo Strandberg, Eva Topinkova, Alfonso J. Cruz-Jentoft, Carlo Custodero, Stefania Maggi, on behalf of the EUROSAF Study Investigators

**Affiliations:** a Geriatrics Unit, Department of Geriatric Care, OrthoGeriatrics and Rehabilitation, E.O. Galliera Hospital, Genova, Italy; b Department of Interdisciplinary Medicine, University of Bari “Aldo Moro”, Bari, Italy; c Geriatrics Unit, Department of Internal Medicine, University of Palermo, Palermo, Italy; d Ageing Clinical Research, Department II of Internal Medicine and Center for Molecular Medicine Cologne, University of Cologne, Faculty of Medicine and University Hospital Cologne, Cologne, Germany; e University of Helsinki and Helsinki University Hospital, Helsinki, Finland; f First Faculty of Medicine, Charles University in Prague, Czech Republic; g Servicio de Geriatría, Hospital Universitario Ramón y Cajal (IRYCIS), Madrid, Spain; h National Research Council, Neuroscience Section, Padova, Italy

**Keywords:** EUROSAF, anticoagulants, frailty, older people, multidimensional prognostic index, comprehensive geriatric assessment

## Abstract

**Background:**

Literature suggests that different risks of mortality could influence physicians in prescribing or not anticoagulants in older patients with atrial fibrillation (AF). The Multidimensional Prognostic Index (MPI) can be considered a tool for the detection of multidimensional frailty. The aim of this cross-sectional study was to evaluate whether prescription patterns of oral anticoagulants exist, based on MPI values.

**Methods:**

Older hospitalised patients (age ≥ 65 years) with non-valvular AF were included across 24 European centres. MPI was calculated using validated and standardised tools derived from a comprehensive geriatric assessment. Other functional and clinical information were collected to calculate indexes specific for haemorrhagic and thromboembolic risk in AF.

**Results:**

Altogether, 2,012 participants affected by AF (mean age was 83.2 ± 7.5, range: 65–104 years), with a higher presence of women (57.0%), were included. Overall, 440 took vitamin K antagonists VKAs (22.0%), 667 (33.4%) direct oral anticoagulants (DOACs), whilst 44.6% did not take any anticoagulant treatment. Prescription of anticoagulants was associated with MPI values, with people taking anticoagulants having lower mean MPI values. Anticoagulant therapy was not used in 53.1% of the group with the highest risk of mortality, compared with 32.3% of those in the group with the lowest mortality risk. People with higher scores in MPI were less frequently treated with anticoagulant therapy, after adjusting for several potential confounders.

**Conclusions:**

The EURopean study of Older Subjects with Atrial Fibrillation (EUROSAF) suggested that almost half of the older persons with AF do not receive anticoagulants and that MPI is an important determinant in prescribing or not anticoagulants. **Trial Registration**: https://clinicaltrials.gov/ct2/show/NCT02973984

**KEY POINTS**
- Atrial fibrillation is a common condition in older people. The data regarding the use of anticoagulants is mainly derived from randomised controlled trials that do not include a sufficient number of older frail people.
- Our study suggests that a consistent part of older people affected by atrial fibrillation was not treated with anticoagulants, in particular, older frail patients; however, it is unclear if this choice is supported or not by evidence.
- The prognostic evaluation through the multidimensional prognostic index could be useful information for the choice in the prescription of anticoagulants in older people affected by atrial fibrillation.

## Introduction

Atrial fibrillation (AF) is a common condition in older people. It is estimated that its prevalence varies from less than 1% in people younger than 50 years to 10%–17% of those aged 80 years or older [1]. It is widely known that oral anticoagulant therapy is effective in preventing stroke and reducing mortality rates in patients with AF [2]. Unfortunately, the use of anticoagulant therapy in clinical practice still remains a challenge in older patients [3], in whom the rate of oral anticoagulant prescribing is often less than 50% [4]. At the same time, the randomised controlled trials (RCTs) leading to the use of direct oral anticoagulants (DOACs) excluded many frail older individuals, that are, on the contrary, among the most affected by AF [5]. Thus, post-marketing studies are urgently needed to further inform physicians on the effectiveness and safety of oral anticoagulants in a “real-world setting,” especially in high-risk, frail or disabled older patients [6].

To better evaluate the benefits and risks of pharmacological and non-pharmacological treatments in older individuals, many guidelines recommend incorporating life expectancy tools and a multidimensional approach into clinical decision-making [7]. In this regard, the Multidimensional Prognostic Index (MPI) is a widely used prognostic index for estimating both short- (in hospital and one month [8] and long-term mortality (from one [9], three [10], five [11] as long as to fifteen [12] years) based on information gathered from a comprehensive geriatric assessment (CGA) [8]. Initially developed and validated in hospitalised older people [8], a series of large multicenter studies including more than 60,000 older subjects across different settings and medical conditions reported that the MPI is an accurate and well-calibrated tool for predicting mortality and other negative health outcomes, including institutionalisation, hospitalisation, re-hospitalization [13], showing high performance in terms of validity, reliability and feasibility for the management of older persons [14].

Regarding anticoagulant therapy, one retrospective observational study including 1,827 community-dwelling older persons affected by AF reported that patients with higher MPI values (indicating a higher presence of multidimensional frailty) were generally less treated with oral anticoagulants than their counterparts [10]. However, a significant benefit given by oral anticoagulant therapy, in terms of mortality reduction, was similar across older people at different prognosis levels [10]. Other data are requested for confirming these observations, particularly because DOACs are rapidly changing prescription practices in many countries [15].

Given this background, the main objective of this cross-sectional work in the context of the EURopean Study of Older Subjects with Atrial Fibrillation (EUROSAF) [15] is to evaluate the anticoagulant prescription patterns in older hospitalised AF European patients and the secondary to evaluate whether MPI score is related to the anticoagulant prescription.

## Materials and methods

The study protocol was previously registered on ClinicalTrials.gov (https://clinicaltrials.gov/ct2/show/NCT02973984). Other details are reported at https://www.eurosaf.eu/home.html.

### Study population and inclusion criteria

EUROSAF is an international, multicenter, prospective, observational study involving elderly subjects (defined as those aged ≥ 65 years [16]) affected by AF hospitalised in 24 European geriatric centres from twelve European countries (Austria, Belgium, Czech Republic, Finland, France, Germany, Italy, Poland, Portugal, Slovakia, Spain, The Netherlands). The study falls in the frame of the Special Interest Group on CGA of the EuGMS (European Geriatric Medicine Society).

All consecutive patients admitted to the Geriatrics Units involved in the project were screened for inclusion. The inclusion criteria were: patients of both genders, aged 65 years and older; admitted to hospital for acute diseases or a relapse of chronic diseases; with a documented diagnosis of non-valvular AF; who are willing to participate in the study and give their informed consent. Exclusion criteria: age less than 65 years; patients not able to provide informed consent; deceased during hospitalisation. The enrolment period lasted from 01^st^ January 2016 to 31^st^ December 2020.

The ethical committees of each centre approved this study. Written informed consent was given by participants who underwent initial evaluation and/or their proxies for their clinical records to be used in this study. All patient records and information were anonymized and de-identified prior to the analysis.

### The multidimensional prognostic index (MPI)

The MPI was calculated at hospital discharge from information obtained through a standard CGA that considered the following eight different domains [8]:

1. Functional status is evaluated by Katz’s Activities of Daily Living (ADL) index [17], which defines the level of dependence/independence in six daily personal care activities (bathing, toileting, feeding, dressing, urine and bowel continence and transferring in and out of bed or chair);
2. Independence in Lawton’s Instrumental Activities of Daily Living (IADL) [18] which assesses independence in eight activities that are more cognitively and physically demanding than ADL, i.e. managing finances, using the telephone, taking medications, shopping, using transportation, preparing meals, doing housework and washing;
3. Cognitive status through the Short Portable Mental Status Questionnaire (SPMSQ) [19], a ten-item questionnaire investigating orientation, memory, attention, calculation, and language; validated versions were used in each local language.
4. Co-morbidity was examined using the Cumulative Illness Rating Scale (CIRS) [20]. The CIRS uses a 5-point ordinal scale (score 1–5) to estimate the severity of pathology in each of 13 systems, including cardiac, vascular, respiratory, eye-ear-nose-throat, upper and lower gastrointestinal, hepatic, renal, genitourinary, musculoskeletal, skin disorder, nervous system, endocrine-metabolic and psychiatric behavioural disorders. Based on the ratings, the Comorbidity Index (CIRS-CI) score, which reflects the number of concomitant diseases, was derived from the total number of categories in which moderate or severe levels (grade from 3 to 5) of disease were identified (ranging from 0 to 13).
5. Nutritional status was investigated with the Mini Nutritional Assessment (MNA) short form (SF) [21], a brief questionnaire comprising anthropometric measurements combined with a questionnaire regarding loss of appetite, recent weight loss, mobility, acute distress, and neuropsychological problems.
6. The risk of developing pressure sores was evaluated through the Exton Smith Scale (ESS), a five items questionnaire determining the physical and mental condition, activity, mobility and incontinence [22].
7. Number of medications taken daily. Medications were categorised using the Anatomical Therapeutic Chemical (ATC) codes.
8. Cohabitation status categorised as living alone, in an institution, or with family members.

For each domain, a tripartite hierarchy was used, i.e. 0 = no problems, 0.5 = minor problems, and 1 = major problems, based on conventional cut-off points derived from the literature for each item. The sum of the calculated scores from the eight domains was divided by 8 to obtain a final MPI risk score ranging from 0 = no risk to 1 = higher risk of mortality [13]. Traditionally, the division of MPI is made using three categories, i.e. MPI-1 (low risk of mortality) <0.33; MPI-2 (intermediate risk) between 0.33 and 0.66; and MPI-3 (high risk) with an MPI value >0.66. Nowadays, MPI is considered a good indicator of multidimensional frailty [23,24]. Therefore, we can consider those with an MPI < 0.33 as robust, between 0.33 and 0.66 pre-frail, and >0.66 as frail [23].

At the following address: multiplat-age.it/index.php/en/tools, it is possible to download for free the software for Windows to calculate the MPI and the tests in different languages including English. The MPI score was available to all prescribing physicians that were adequately trained, or they have experience in using MPI in hospitals.

### Anticoagulants’ prescription

Participants were divided into three categories according to the prescription of anticoagulants. Using the ATC codes, vitamin K antagonists (VKAs) included warfarin, acenocumarol, dicoumarol, phenindione, whilst DOACs included dabigatran, rivaroxaban, apixaban, and edoxaban. Participants not taking VKAs or DOACs were categorised as having no treatment.

### Systemic thromboembolic and bleeding risk

Besides the information for calculating the MPI, we also collected the systemic thromboembolic risk by using the CHA2DS2-Vasc score (congestive heart failure, hypertension, age category, diabetes, stroke, vascular disease, gender) and the bleeding risk by using the HAS-BLED score (hypertension, abnormal liver or renal function, stroke, bleeding, labile INR, old age, drugs or alcohol). Moreover, general information regarding the reasons for which anticoagulants were not prescribed was recorded with open questions. Finally, we also reported the information regarding antiplatelet therapy.

### Statistical analysis

The demographic and clinical characteristics of the patients were reported as mean and standard deviations for continuous variables or frequency and percentage for categorical variables. The normality of the distribution of continuous variables was investigated by using the Kolmogorov–Smirnov test. Between-group comparisons were performed using analysis of variance (ANOVA) for continuous variables and the Pearson Chi-Square test for categorical ones.

We reported a logistic binary regression analysis taking as an outcome the use of anticoagulants or not and as exposures the factors significantly associated in univariate analysis (*p*-value < .10) as results of the comparison of the anticoagulants’ prescription status at the discharge, also considering the Bonferroni’s correction. In order to remove the redundancy of covariates included, we assessed the collinearity of the factors included, setting a variance inflation factor (VIF) of more than two as the reason for exclusion [25]. The discriminative ability of the logistic regression model was assessed using the concordance (c- statistics), an index indicating the probability that a randomly selected subject who experienced the outcome (i.e. the use of anticoagulants) will have a higher predicted probability of having the outcome occur compared to a randomly selected subject who did not experience the event [26]. Finally, single domains of MPI were included in multivariate logistic regression analysis for investigating the importance of single domains of CGA in determining the prescription of anticoagulants in our study.

A *p* value < .05 was considered statistically significant, taking into account Bonferroni’s correction. Therefore, since three groups with three possible comparisons were analysed, we considered a *p*-value < .05/3 (0.017) as statistically significant. All statistical analyses were performed using SPSS software (version 21.0).

## Results

As shown in Supplementary Figure 1, 2,164 participants were initially included. After removing 152 participants since MPI was not calculable (89 deaths during the hospitalisation and 63 with no sufficient information), 2,012 older participants affected by AF (92.3% of the initial population) were analysed. The 152 participants without sufficient information for calculating the MPI did not differ in terms of age and sex, compared to those included. No other salient characteristics were significantly different between the two groups.

The participants included in the study aged a mean of 83.2 years (SD: 7.5, range: 65–104 years) and 57.0% were females. Their mean MPI value was 0.50 ± 0.20. Overall, 526 (26.1%) were classified as MPI 1 (robust), 948 (47.1%) in MPI 2 (pre-frail) and 538 in MPI 3 (26.8%) (frail). As reported in Table 1, VKAs were prescribed to 440 patients (22.0%) and DOACs were prescribed to 667 patients (33.4%), whilst the remaining 44.6% did not take any anticoagulant treatment. Before hospitalisation the previous figures were 28.7% for VKAs, 33.9% for DOACs and 37.3% for those not treated. Among the reasons for which anticoagulant therapy was not given at the discharge, the three most frequent were high risk of falls (17.8% of the answers), previous bleeding (15.8%) or high HAS-BLED score (12.6%), indicating a potential future high risk of bleeding. Both VKAs and DOACs were prescribed more frequently to people with lower MPI values (*p* < .0001 for both VKAs and DOACs versus no anticoagulants). Among the individual domains of the MPI the risk of pressure sores, the presence of disabilities in basic and instrumental activities of daily living, the higher number of comorbidities, and a poor nutritional status were associated with different approaches in anticoagulants’ prescription, whilst cognitive status, the number of drugs and co-habitation status were not associated with the use of anticoagulants (Table 1). No significant differences between people treated with VKAs and DOACs were reported in any of the domains analysed. The participants for which no anticoagulant was prescribed took more frequent antiplatelet therapy compared to VKAs and DOACs.

**Table 1.** Baseline characteristics by anticoagulant therapy.

Parameter	No anticoagulant treatment (*n* = 905)	VKAs (*n* = 440)	DOACs (*n* = 667)	*p*-value VKAs vs. no anticoagulant treatment	*p*-value DOACs vs. no anticoagulant treatment	*p*-value DOACs vs. VKA
Age	83.3 ± 7.5	83.5 ± 7.6	82.8 ± 7.4	.43	.39	.08
Females (%)	54.9	57.0	59.9	.08	.07	.99
CHA2DS2-VASC	4.9 ± 1.5	4.8 ± 1.4	4.9 ± 1.5	.08	1.00	.41
HAS-BLED	3.0 ± 1.2	2.6 ± 1.0	2.6 ± 1.2	<.0001	<.0001	1.00
Antiplatelet therapy (%)	11.8	2.3	3.6	<.0001	<.0001	1.00
SPMSQ	3.2 ± 3.2	2.9 ± 2.9	2.8 ± 2.9	1.00	.34	1.00
ESS	14.8 ± 3.6	16.4 ± 3.1	16.2 ± 3.2	<.0001	<.0001	1.00
ADL	3.3 ± 2.2	4.1 ± 2.1	3.8 ± 2.2	<.0001	<.0001	.78
IADL	3.2 ± 2.7	3.9 ± 2.9	3.9 ± 2.9	.04	<.0001	1.00
CIRS-CI	4.5 ± 2.4	3.6 ± 2.1	4.0 ± 2.2	<.0001	<.0001	.23
MNA-SF	8.8 ± 3.2	10.1 ± 2.9	9.5 ± 3.1	<.0001	<.0001	.06
Number of drugs	4.0 ± 3.5	3.3 ± 2.5	3.5 ± 2.7	1.00	1.00	1.00
Alone (%)	34.2	31.5	33.8	.56	.55	.94
MPI	0.54 ± 0.20	0.46 ± 0.20	0.48 ± 0.21	<.0001	<.0001	1.00
MPI 1 (%) <0.33	19.0	32.4	31.6	<.0001	.19	.04
MPI 2 (%) 0.34–0.66	48.7	49.1	43.7	.99	1.00	.78
MPI 3 (%) >0.66	32.3	18.6	24.7	<.0001	.01	.97

Abbreviations: MPI: multidimensional prognostic index; ADL: activities of daily living; IADL: instrumental activities of daily living; SPMSQ: short portable mental state questionnaire; ESS: Exton-Smith Scale; MNA-SF: Mini Nutritional Assessment-Short Form; CIRS-CI: Cumulative Illness Rating Scale-Comorbidity Index; CHA2DS2-VASC: congestive heart failure, hypertension, age category, diabetes, stroke, vascular disease, sex category; HAS-BLED. hypertension, abnormal liver or renal function, stroke, bleeding, labile INR, old age, drugs or alcohol.

Table 2 shows the logistic regression analysis taking anticoagulant therapy (VKAs and DOACs) versus no anticoagulant therapy as an outcome. The c-statistics for the model including age, sex, HAS-BLED, CHA2DS2-VASC, the use of anti-platelet medications and MPI was 0.66 (95%CI: 0.63–0.68; *p* < .0001), compared 0.63 (95%CI: 0.60–0.65; *p* < .0001) of the model without MPI. People in MPI 2 and MPI 3 were less frequently treated with anticoagulant therapy (OR = 0.50; 95%CI: 0.39–0.64; *p* < .0001 for MPI 2 and OR = 0.37; 95%CI: 0.28–0.50; *p* < .0001) compared to their counterparts in MPI 1 and after adjusting for age, sex, HAS-BLED, CHA2DS2-VASC and the use of anti-platelet medications (Table 2). Among the single domains of MPI (Supplementary Table 1), higher scores of MNA and SPMSQ, indicating a better nutritional and cognitive status, respectively, were associated with a higher prescription rate of anticoagulants, whilst higher CIRS levels and the number of medications were associated with a lower prevalence of anticoagulants’ prescription.

**Table 2.** Multivariate logistic regression analysis using anticoagulant therapy as outcome and factors of interest as exposures.

Factor	OR	95% CI low	95% high	*p*-value
MPI 1	Reference			
MPI 2	0.497	0.389	0.635	<.0001
MPI 3	0.373	0.280	0.497	<.0001
Age	1.006	0.993	1.020	.350
Male gender	1.066	0.871	1.303	.536
HAS-BLED	0.728	0.664	0.797	<.0001
CHA2DS2-VASC	1.144	1.062	1.232	<.0001
Use of anti-platelet medications	0.234	0.155	0.353	<.0001

**Figure 1.**
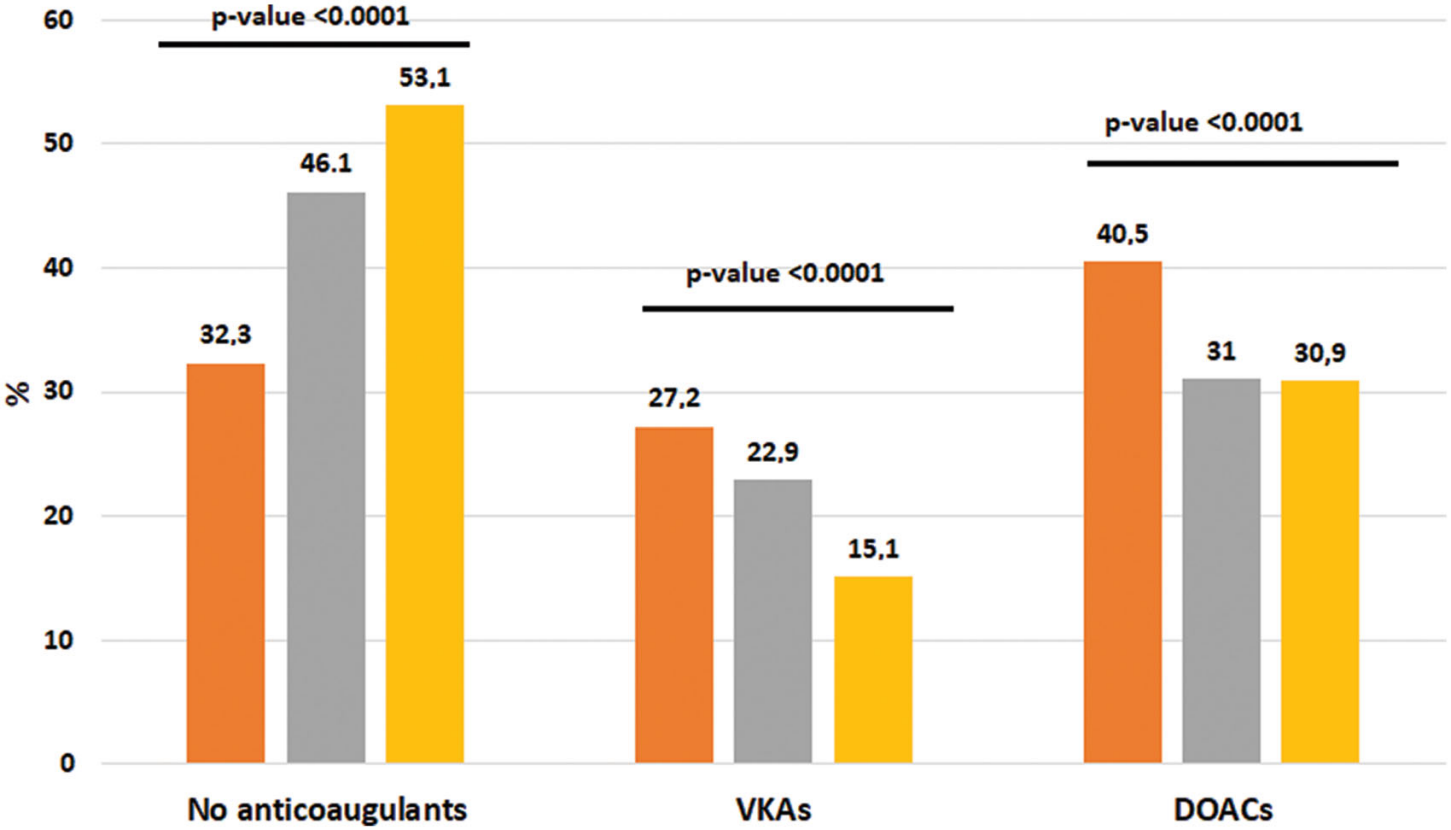
Anticoagulant prescription by multidimensional prognostic index values. Legend: Participants in MPI 1 are orange, MPI 2 grey, and MPI 3 yellow. P-values referred to the percentage of participants with different MPI values for each anticoagulant category (no anticoagulants, vitamin K antagonists (VKAs), direct oral anticoagulants (DOACs).

Figure 1 shows the rate of anticoagulant prescriptions according to the MPI classes. Overall, 44.6% of older patients with AF were not prescribed anticoagulant therapy, while 22.0% of them were prescribed VKAs and 33.4% were prescribed DOACs (*p* < .0001). As shown in Figure 1, a significant progressive decrease in the prescription of anticoagulants was observed in AF older patients with the increasing of their MPI class of risk: anticoagulant prescriptions in MPI-1 class (indicating robust participants) 67.7%, vs MPI-2 53.9% vs MPI-3 class (frailer participants) 46.9% (*p* for trend < .0001). Moreover, physicians prescribed more frequently in people in MPI-3 (those at highest risk) DOACs (30.9%) than VKAs (15.1%), even if more than half of these participants did not receive any prescription for anticoagulant therapy (*p* < .0001) (Figure 1).

## Discussion

The EUROSAF included more than 2,000 older hospitalised people affected by AF enrolled across several European countries and centres. We believe that the participants, ageing in mean of 83 years, reflect the patients that in daily clinical practice had the highest prevalence of AF [1]. Overall, our study indicates that the prescription of anticoagulants could be dependent also on MPI values, other than other factors usually associated with the prescription or not of anticoagulants’ treatment, therefore highlighting that the presence of multidimensional frailty could be associated with the prescription of anticoagulants in older people affected by AF. We believe that the results of this cross-sectional analysis could be useful for physicians involved in the care of older people.

It is important to note that about half of older people did not take any anticoagulant therapy at hospital discharge, confirming previous reports of a sub-optimal prescription of oral anticoagulants in older with AF [27]. The rate of older people for which anticoagulant therapy is not prescribed remains high despite evidence of increased benefit in these patients [28]. Theoretically, older patients should receive anticoagulant therapy for AF such as younger individuals, in the case of males with a CHA2DS2-VASc score ≥2 and females with a score ≥3 [2]. In our sample, however, only a limited part of the participants included had no clinical indication for anticoagulant therapy (among males 10/864, among females only 8 over 1148) indicating that the large majority should be treated with anticoagulants. However, VKAs were traditionally underused in older people, fearing that this subgroup of patients would have higher side effects due to non-adherence to INR monitoring and drug use [13]. At the same time, the data from the real-world setting showed a mis-prescription of DOACs in particular among frail older adults, also because the regulatory RCTs failed to include the geriatric population [5]. Our study suggests that in older people included in the MPI 3 group, i.e. participants that we can consider frail, the prescription rate of DOACs is practically doubled than VKAs. These data suggest that if physicians decide to prescribe anticoagulants in geriatric frail patients, they prefer DOACs over VKAs. This is justified for several reasons, including no need for INR (International Normalised Ratio) monitoring or lower risk of haemorrhagic events, compared to VKAs. Moreover, the greater prescription of DOACs vs VKAs in the MPI 3 group may indicate that physicians are changing their prescription patterns guided by the extensive evidence generated in the last ten years, overall indicating that DOACs are safer, particularly in terms of intracranial haemorrhagic events, and at least as effective as VKAs for thromboembolic prophylaxis in AF in older and frail populations [29]. However, in frailer patients, the undertreatment with anticoagulants is still present and probably due to factors such as limited life expectancy, risks related to the treatment, contraindications, multimorbidity, and polypharmacy [30].

Anticoagulant therapy is an important topic in geriatric medicine, introducing the importance of clinical-decision making tools [31]. In literature, the main reasons reported to refrain from the prescription of oral anticoagulant therapy in older people include age itself, current concomitant antiplatelet therapy, an increased risk of bleeding or risk of falls, cognitive impairment, functional impairment or difficulty in maintaining adequate INR values [32]. By contrast, given the lack of specific recommendations, the potential opposite risk is the prescription of such drugs only based on stroke risk scores and not accounting frailty condition of older adults with AF [33].

Many studies suggested that age is an important determinant of the prescription of anticoagulants [34]. Another important finding of the EUROSAF is that mean age was not significantly different between people taking VKAs or DOACs and those not taking any anticoagulant, indicating that “age is just a number” and that age alone seems not to influence prescriber decision to treat AF with anticoagulants, being only a marker of other determinants (e.g. diseases, disability) [35]. On the contrary, MPI values were significantly higher in people not taking anticoagulants indirectly reflecting the fact that other determinants detected through multidimensional assessment may influence treatment more than age *per se,* even if the cross-sectional nature of our work does not permit to explain a cause-effect association [31]. In this sense, the EUROSAF study reports that in more than half of people in MPI-3 group, indicating people at higher risk of an unfavourable prognosis, no anticoagulant therapy was prescribed compared to about one third of more robust people. Altogether, these findings suggest that a different risk of mortality, as indicated by the MPI, may influence the attitude of physicians in prescribing oral anticoagulants in older people with AF, potentially indicating that the MPI-based prognostic information can be useful to physicians in identifying older patients with AF that can benefit from oral anticoagulant treatment in terms of prolonged survival [15].

The choice not to give anticoagulants to older people seems to be attributable to several reasons, also based on the literature regarding this topic. First, as also confirmed in the EUROSAF study, the risk of falls and previous bleedings are probably the two most important reasons for not giving any anticoagulant therapy, even if are not formal contraindications to anticoagulant therapy. Second, the literature suggests that polypharmacy, multimorbidity and the presence of some frequent conditions, such as dementia, can discourage physicians from prescribing anticoagulants [4,36]. However, the EUROSAF study suggests that among the single domains of MPI, a better nutritional and cognitive status, respectively, were associated with a higher prescription rate of anticoagulants, whilst comorbidities and number of medications were associated with a lower prevalence of anticoagulants’ prescription. Of importance, no one of the single domains remained statistically significantly associated with anticoagulants’ prescription, overall indicating that multidimensional frailty is more important than the single domains, also in this kind of patient.

For all these reasons, we believe that the correct management of anticoagulant therapy in older people may depend on the presence of multidimensional frailty, as assessed by MPI [10,31]. Even if the presence of frailty is of critical importance for anticoagulants’ prescription, the presence of a multidimensional assessment, derived from a CGA, is usually not included in the decision algorithms of the most appropriate treatments [37]. We believe that findings from the EUROSAF study will be reflected in future AF guidelines recommending frailty assessment and prognostic information be included to maximise the benefits of anticoagulant therapy. In this sense, for example, one-third of the people in MPI-1 were not treated and this indicates the baseline ageist attitude of physicians in this regard.

The findings of our study must be considered within its limitations. First, the cross-sectional nature of this analysis may prevent a generalisation of these data. Second, MPI was calculated only at discharge: therefore, people who died during hospitalisation that could be frailer were not included and this could introduce a selection bias. For example, at admission, only 37.3% were without anticoagulant, but it seems that quite a several VKA users stopped its use during the hospitalisation and were discharged with any anticoagulant treatment. Unfortunately, it is unclear to which MPI group those who stopped the use belonged to since no information regarding this factor was present at the admission. Third, there is no randomisation to understand the impact of the MPI on anticoagulation prescription, and as such the impact of MPI and treatment patterns on outcomes could be not established. Finally, the participants were recruited during hospitalisation, mainly in geriatric wards and we did not collect either the number of patients with atrial fibrillation screened and the reasons for exclusion and so they could represent a selected sample possibly introducing a selection bias.

In conclusion, the data of the cross-sectional analysis of the EURopean study of Older Subjects with Atrial Fibrillation (EUROSAF) suggested that many older people are not treated with anticoagulants, despite their potential benefit. MPI could be an important determinant in prescribing or not anticoagulants, further indicating the necessity of using a CGA-derived tool for better approaching AF in older people. The longitudinal data of the EUROSAF study will indicate if anticoagulants are safe and effective in older people and if MPI can indicate people with different mortality risks have benefited from anticoagulant therapy.

## Supplementary Material

Supplemental Material

Supplemental Material

## Data Availability

The data underlying this article will be shared on reasonable request to the corresponding author.

## EUROSAF Investigators:

The EUROSAF Study Investigators includes:

1. Alberto Ferri, Alessandra Argusti, Federica Gandolfo, Clarissa Musacchio, Katerin Leslie Quispe Guerrero, Alberto Pilotto, Geriatrics Unit, Department of Geriatric Care, OrthoGeriatrics and Rehabilitation, E.O. Galliera Hospital, Genova, Italy
2. Carlo Custodero, Vincenzo Solfrizzi, Carlo Sabbà, Department of Interdisciplinary Medicine, University of Bari “Aldo Moro”, Bari, Italy
3. M.Cristina Polidori^a,b^, Joshua Verleysdonk^a^, Nico Noetzel^a^, ^a^Ageing Clinical Research, Department II of Internal Medicine and Centre for Molecular Medicine Cologne, University of Cologne, Faculty of Medicine and University Hospital Cologne, Cologne, Germany – ^b^Cluster of Excellence CECAD, University of Cologne, Cologne, Germany
4. Timo Strandberg, Juhani Rossinen, Laura Pikkarainen, Helsinki University Hospital; City of Helsinki, Social Services and Health Care Division, Finland
5. Tuomo Nieminen, South Karelia Central Hospital, Dept. Internal Medicine, Eksote, Lappeenranta, Finland; Päijät-Häme Joint Authority for Health and Wellbeing, Finland
6. Eva Topinkova, Helena Michalkova, Pavla Madlova, Lucie Bautzka, Department of Geriatric, 1st Faculty of Medicine, Charles University and General University Hospital (GUH), Prague, Czech Republic
7. Stefania, Maggi, National Research Council, Neuroscience Section, Padova, Italy
8. Nicola Ferrara, Lucia Gioia, Anna Maria Iannicelli, Department of Traslational Medical Sciences, Federico II^^^ University, Napoli, Italy
9. Mario Barbagallo, Nicola Veronese, Giovanna Di Bella, Geriatrics Unit, Department of Internal Medicine, University of Palermo, Palermo, Italy
10. Giovanni Ruotolo, Alberto Castagna, A.O. Pugliese-Ciaccio, SOC di Geriatria Geriatrics Unit, Ciaccio Hospital Catanzaro, Italy
11. Regina Roller-Wirnsberger, Christian Sebesta, Sonja Lindner, Wiener Krankenanstaltenverbund Sozialmedizinisches Zentrum Ost Donauspital, Vienna, Austria
12. Alfonso Cruz-Jentoft, Luisa A. Hernández-Sánchez, Jana Albéniz López, Génesis Estefanía Olaya-Loor, Servicio de Geriatria, Hospital Universitario Ramon y Cajal (IRYCIS), Madrid, Spain
13. Pedro Marques da Silva (*), Hospital Santa. Marta-Centro Hospitalar de Lisboa Central, Núcleo de Investigação Arterial, Medicina 4, Lisboa, Portugal
14. Heidi Gruner, Centro Hospitalar de Lisboa Central, EPE – CHLC, Hospital de Curry Cabral, Lisboa, Portugal
15. Jean Petermans (*), Sophie Gillain, Véronique Jonart, CHU Liege, Geriatrics Department, University Hospital of Liège, Belgium
16. Ondrej Vyska, Jiri Nakladal, Katarina Bielakova, Hana Matejovska-Kubesova, University Hospital Brno, Dept. Internal Medicine – Geriatrics and General Practice, Brno, Czech Republic
17. Adrian Enica, Stéphanie Roth, Benjamin Jacquet, Vito Curiale, Rainer III Gerontologic Centre, Princess Grace Hospital, Principality of Monaco
18. Nicolas Berg, Livia Mirea Cimpeanu, Service de Geriatrie, CHR de la Citadelle, Liege, Belgium
19. Rafaela Verissimo, Leonor Silva, Luciana Silva, Pedro Magalhães, Orthogeriatric Unit and Geriatric Medicine Ward, Department of Internal Medicine – Centro Hospitalar de Vila Nova de Gaia/Espinho, Portugal
20. Gabriel Ioan Prada, Anna Marie Herghelegiu, Catalina Raluca Nuta, "Carol Davila" University Of Medicine and Pharmacy Bucharest, Romania and "Ana Aslan" Institute of Gerontology and Geriatrics, Bucharest, Romania
21. Blanca Garmendia-Prieto, Isabel Lozano-Montoya, Javier Jaramillo-Hidalgo, Javier Gómez-Pavón, Servicio de Geriatría, Hospital Central de la Cruz Roja “San José y Santa Adela”, Facultad de Medicina, Universidad Alfonso X El Sabio, Madrid, Spain
22. Ursula Müller-Werdan, Gordon Werth, Adrian Rosada Department of Geriatrics and Medical Gerontology, Charité – Universitätsmedizin Berlin
23. Ozlem Yilmaz, Sena Gürkaş, Istanbul University, Faculty of Istanbul Medicine, Geriatric Unit, Istanbul, Turkey
24. Francesco Mattace-Raso, Harmke Polinder-Bos, Division of Geriatric Medicine, Department of Internal Medicine, Erasmus MC University Medical Centre, Rotterdam, The Netherlands
