# Supplementary material for: The role of prognostic stratification on prescription of anticoagulants in older patients with atrial fibrillation: a multicenter, observational, prospective European study (EUROSAF)
Source: Ann Med. 2022 Sep 5;54(1):2411–9. doi: 10.1080/07853890.2022.2117407 (PMC9448408; doi:10.1080/07853890.2022.2117407)
Supplement: Supplemental Material [file IANN_A_2117407_SM2621.docx]

**Supplementary Table 1. Results of logistic regression analysis including the single domains of the multidimensional prognostic index, with anticoagulants’ prescription as outcome.**

| **Domains** | **Odds ratio (95% confidence intervals)^1^** | **P-value** |
| --- | --- | --- |
| **ADL** | 1.00 (0.92-1.08) | 0.94 |
| **IADL** | 1.00 (0.95-1.06) | 0.86 |
| **MNA-SF** | 1.05 (1.009-1.09) | 0.02 |
| **SPMSQ** | 1.07 (1.02-1.11) | 0.003 |
| **CIRS-CI** | 0.94 (0.89-0.98) | 0.009 |
| **ESS** | 1.14 (1.09-1.20) | <0.0001 |
| **Number of drugs** | 0.94 (0.89-0.99) | 0.02 |
| **Living with family** | 1.11 (1.02-1.21) | 0.01 |

^1^ The results are reported as odds ratios (ORs) with their 95% confidence intervals (CIs) and p-values for each domain of the multidimensional prognostic index, all as continuous, adjusted for age, sex, CHA2DS2-VASC and HAS-BLED. Values of the ORs >1 indicate a higher prescription rate of anticoagulants, <1 a lower.

**Abbreviations:** ADL: activities of daily living; IADL: instrumental activities of daily living; SPMSQ: short portable mental state questionnaire; ESS: Exton-Smith Scale; MNA-SF: Mini Nutritional Assessment-Short Form; CIRS-CI: Cumulative Illness Rating Scale-Comorbidity Index; CHA2DS2-VASC: congestive heart failure, hypertension, age category, diabetes, stroke, vascular disease, sex category; HAS-BLED. hypertension, abnormal liver or renal function, stroke, bleeding, labile INR, old age, drugs or alcohol.
