# Supplementary figures and images for: The role of prognostic stratification on prescription of anticoagulants in older patients with atrial fibrillation: a multicenter, observational, prospective European study (EUROSAF)
Source: Ann Med. 2022 Sep 5;54(1):2411–9. doi: 10.1080/07853890.2022.2117407 (PMC9448408; doi:10.1080/07853890.2022.2117407)

## Slide 1
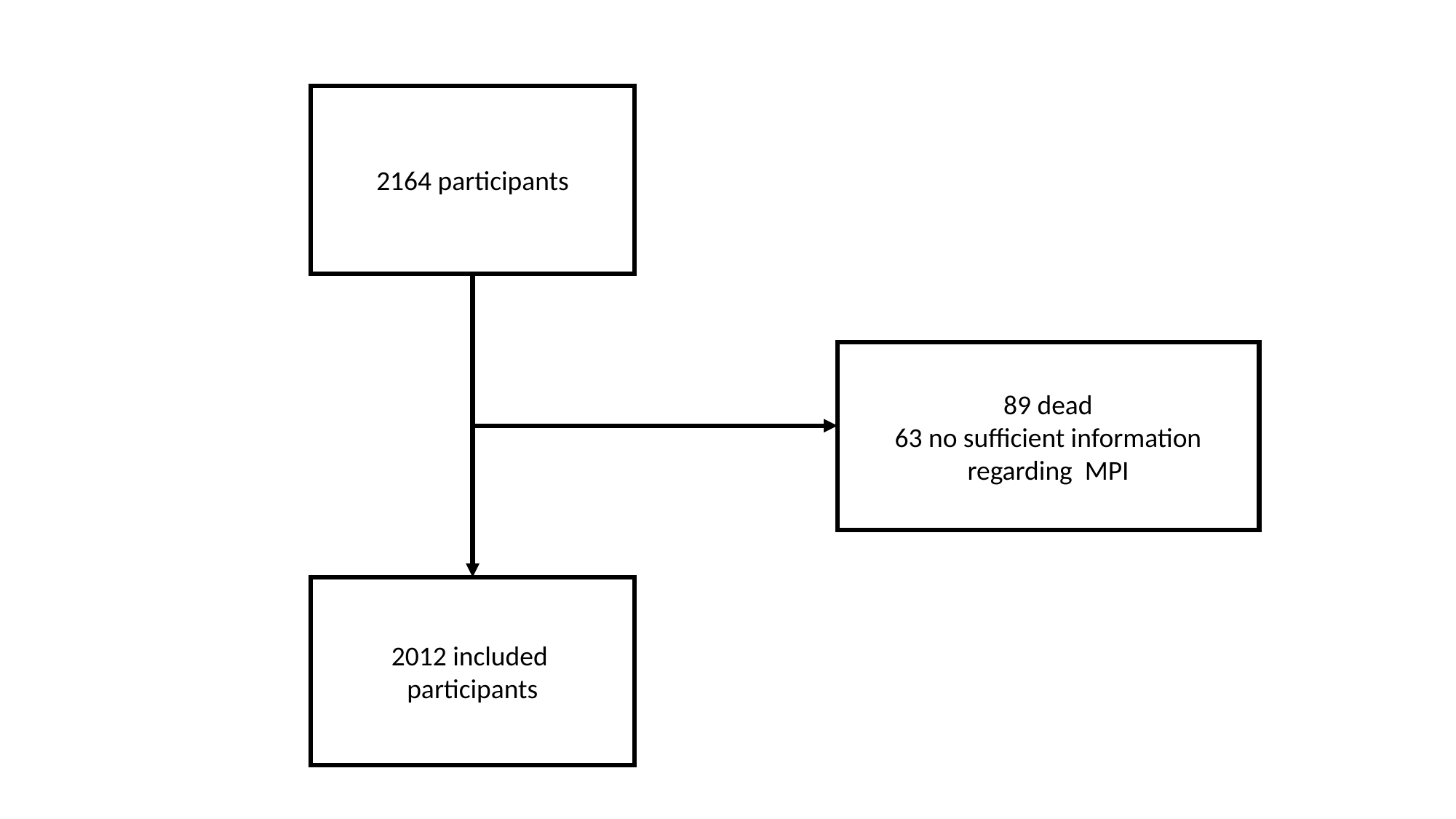

2164 participants
89 dead
63 no sufficient information regarding MPI
2012 included
participants

Supplement: Supplemental Material [file IANN_A_2117407_SM2619.pptx]
